# Our evolving understanding of the role of the γδ T cell receptor in γδ T cell mediated immunity

**DOI:** 10.1042/BST20200890

**Published:** 2021-09-13

**Authors:** Benjamin S. Gully, Jamie Rossjohn, Martin S. Davey

**Affiliations:** 1 Infection and Immunity Program and Department of Biochemistry and Molecular Biology, Biomedicine Discovery Institute, Monash University, Clayton, Victoria 3800, Australia; 2 Institute of Infection and Immunity, Cardiff University School of Medicine, Heath Park, Cardiff CF14 4XN, U.K.

**Keywords:** immunology, structural biology, T-cells

## Abstract

The γδ T cell immune cell lineage has remained relatively enigmatic and under-characterised since their identification. Conversely, the insights we have, highlight their central importance in diverse immunological roles and homeostasis. Thus, γδ T cells are considered as potentially a new translational tool in the design of new therapeutics for cancer and infectious disease. Here we review our current understanding of γδ T cell biology viewed through a structural lens centred on the how the γδ T cell receptor mediates ligand recognition. We discuss the limited knowledge of antigens, the structural basis of such reactivities and discuss the emerging trends of γδ T cell reactivity and implications for γδ T cell biology.

## Introduction

The human adaptive immune system has conserved a tripartite lymphocyte compartment comprising B cells, αβ and γδ T cells for over 450 million years of vertebrate evolution [1]. Whilst great strides have been made in B cell and conventional αβ T cell mediated immunity, the functional role of γδ T cells have remained less well defined. Notwithstanding this, γδ T cells are known to play a central, and somewhat unique, role in both anti-microbial [2] and anti-tumour immunity [3], in addition to roles in tissue homeostasis and mucosal immunity, reviewed in [4].

## Current understanding of γδ T cell lineages

**Figure 1.**
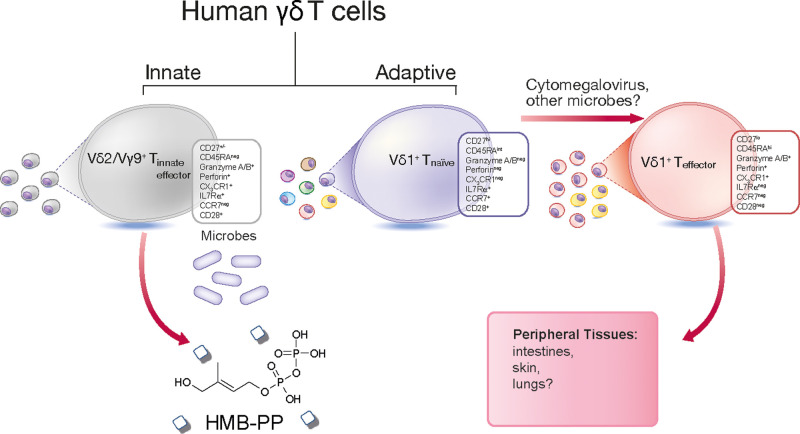
Innate-like and adaptive human γδ T cell sub-compartments. Vγ9/Vδ2^+^ T cells are formed of an innate-like T cell population that possess a semi-invariant T cell receptor (TCR) and reactivity to the microbial-derived metabolite HMB-PP. Whereas post-natal Vδ1^+^ T cells can be separated into naïve-like and effector populations. Naïve-like Vδ1^+^ T cells display a diverse TCR repertoire and express CD27, CD28, CD62L, CCR7 and IL7Rα on their cell surface. In contrast, effector Vδ1^+^ T cells often possess clonally focussed TCRs, express low levels of CD27, up-regulate expression of the endothelial homing receptor CX3C chemokine receptor 1 (CX3CR1) and cytotoxic granzymes and perforin.

Framed by a lack of clarity on complementary ligands γδ T cells are often delineated into subsets based on the expressed Vδ gene segment, namely Vδ1^+^, Vδ2^+^ and Vδ3^+^ subsets. The major peripheral blood subset are Vδ2^+^ γδ T cells, comprising ∼1–10% of circulating T cells [5]. These prototypic unconventional T cells almost exclusively express a Vγ9 chain, resulting in a focused peripheral repertoire at birth that polyclonally expands following postnatal microbial colonisation [6–8]. Thus Vδ2^+^ cells offer both an innate-like prenatal protection mediated by Vδ2^+^Vγ9^+^ T cells that persist into adulthood alongside an adaptive-like immunobiology of Vδ2^+^Vγ9^−^ T cells that clonally expand upon cytomegalovirus infection and exhibit effector function [9]. Vδ2^+^Vγ9^+^ T cells play a central role in the rapid anti-microbial immune response to bacteria, [10] parasites, [11] and tumour cells (Figure 1) [12,13].

Vδ2^−^ γδ T cells, encompassing Vδ1^+^ and Vδ3^+^ populations, have been implicated in anti-viral immunity to cytomegalovirus [14,15] and Epstein Barr virus [16,17] as well as potential roles in tumour surveillance [18]. Vδ1^+^ T cells are the most abundant neonatal γδ lineage and continue as the predominating subset in adult peripheral tissues including the gut, skin and liver [19–22]. Although Vδ1^+^ T cells can utilise germline encoded innate receptors, namely NKG2D, NKp30 and NKp46, [23–25] our understanding of this lineage is far from complete, with recent identification of foetal population of invariant Vδ1^+^ T cells that precede the development of a postnatal naïve-like and effector Vδ1^+^ T cell sub-compartments, dependent on T cell receptor (TCR) selection and eluding towards distinct adaptive-like properties (Figure 1) [26–29].

The role of Vδ3^+^ γδ T cells has largely eluded understanding with early insights into immunobiology finding Vδ3^+^ T cells comprise a population of gut intraepithelial lymphocytes (IEL) [30]. Vδ3^+^ T cells also form a discrete population (∼0.2%) of CD4^−^CD8^−^ peripheral T cells that are postulated to be innate-like due to the expression of NKG2D, CD56 and CD161 [31]. Peripheral Vδ3^+^ frequencies are known to increase in lupus patients, [32,33] cytomegalovirus [14] and HIV [34] although our knowledge of their immunobiology remains incomplete.

## Current understanding of γδ T cell activation

While bifurcation of γδ T cells by Vδ gene usage has revealed much about their biology, questions remain about antigen reactivity. Indeed, a central tenet of γδ T cell biology has been their suggested breadth of antigen reactivity. The majority of bona fide ligands identified for human γδ T cells to date have been described for the Vδ1 lineage, which can recognise proteins that adopt the MHC-like fold CD1b, [35] CD1c, [36] CD1d [37,38] and MR1 [39]. Broader Vδ1 reactivity towards non-MHC-like proteins has been established for Phycoerythrin, [40] and more recently Ephrin type-A receptor 2 [41] alongside Vδ3 and Vδ5 reactivity towards Annexin A2 and endothelial coupled protein-C receptor, respectively (Table 1) [42,43].

**Table 1:** Identified γδ TCR ligands and gene restrictions

Human γδ lineage	Tissue	Ligand	Structural fold	Ref.
Vδ restriction				
*Vδ1*	Peripheral blood	CD1b	MHC class I–like	[35]
*Vδ1*	Peripheral blood	CD1c	MHC class I–like	[36]
*Vδ1*	Peripheral blood	CD1d	MHC class I–like	[37,38]
*Vδ1/Vδ3*	Peripheral blood	MR1	MHC class I–like	[39]
*Vδ1*	Peripheral blood	R-Phycoerythrin	Phycobiliprotein	[40]
*Vδ3*	-	Annexin A2	Annexin	[43]
Vγ restriction (or pairings)				
*Vγ4*	Enteric	BTNL3-BTNL8	B7-like	[71,73,78]
*Vγ9–Vδ2*	Peripheral blood	BTN3A-BTN2A	B7-like	[47,63]
*Vγ3–Vδ2*	Intramuscular	Aminoacyl tRNA synthetase	Class II synthetase	[79]
*Vγ4–Vδ5*	Peripheral blood	Endothelial protein-C receptor	MHC α1-α2–like	[42]
*Vγ9–Vδ1*	Peripheral blood	Ephrin type A receptor 2	Ephrin receptor tyrosine kinase	[41]

Conversely, Vδ2^+^Vγ9^+^ T cells are broadly reactive to phosphoantigens that include (E)-4-Hydroxy-3-methyl-but-2-enyl pyrophosphate (HMBPP) a metabolite of the MEP pathway in Plasmodium falciparum, and isopentenyl pyrophosphate (IPP) a metabolite in the mevalonate pathway [10]. Such phosphoantigen (pAg) reactivity is dependent on the B7-protein like butyrophilin (BTN) 2A1 and 3A1 as well as the TCR (Table 1) [44–46]. The pAg activation of Vδ2^+^ T cells and the nexus of BTN and TCR molecules remains poorly understood despite growing understanding of their central importance in such reactivities [47]. Thus γδ T cell receptors seem to enable recognition of a broader range of protein antigens, compared with their αβ counterparts.

## Structural determinants of γδ T cell reactivity

Clarity around the paradigms for T cell reactivities, and indeed principles of selection, have been greatly advanced through a structural lens. Here, a multitude of αβ TCR structures have solidified our understanding of MHC-restriction, T cell immunity and autoreactivities for conventional and unconventional αβ T cells. In contrast, a dichotomy around δ T cell reactivities has provided confusion, here parallels are often drawn to conventional T cell subsets, yet a defining feature of γδ T cells is the diversity of the antigens they recognise and the suggestion of how such reactivities are achieved.

**Figure 2.**
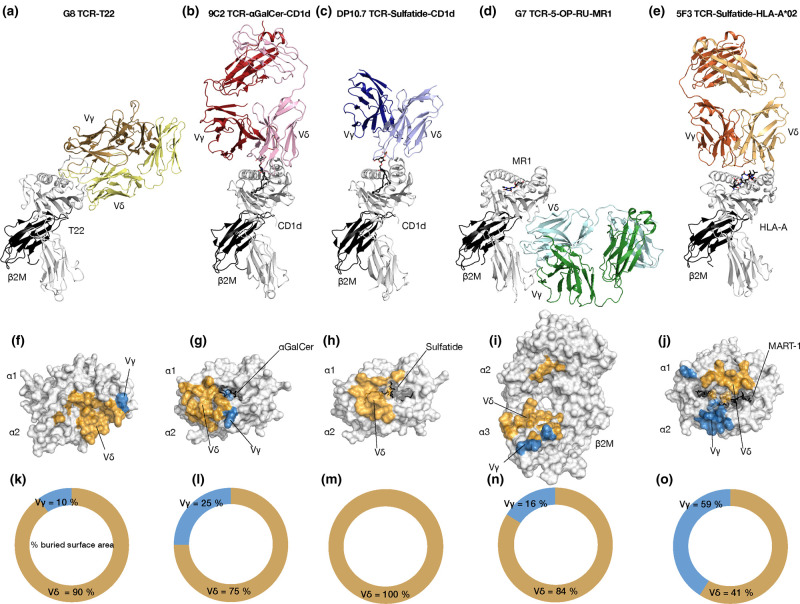
Analysis of the Vδ dominated interface of MHC-like molecule reactivity. Overall docking mode and interface analysis of yd TCR-antigen ternary structures. Cartoon representation of the Murine G8 (Vγ4Vα11.3) TCR mediated recognition of T22 (**a**), 9C2 (Vγ5Vδ1) TCR mediated recognition of CD1d-aGalCer (**b**), DP10.7 (Vγ4Vδ1) TCR mediated recognition of CD1d-sulfatide (**c**), G7 (Vγ9Vδ1) TCR mediated recognition of MR1-5-OP-RU (**d**) and 5F3 (Vγ8Vδ1) TCR mediated recognition of HLA*A2-MART-1 (**e**). Interface analysis of the corresponding complex TCR structures with the Vδ and Vγ mediated contacts coloured orange and blue, respectively, with the percentage contributions to the BSA shown, G8-T22 (**f** and **k**), 9C2-CD1d-aGalCer (**g** and **l**), DP10.7-CD1d-sulfatide (**h** and **m**), G7-MR1-5-OP-RU (**i** and **n**) and 5F3-HLA*A2-MART-1 (**j** and **o**).

Although this review is focused on human γδ T cells, the first structural insight was provided via comparison to a murine γδ TCR structure in complex with the stress induced β2-microglobulin associated MHC class I molecule, T22. Here, polyclonal T cell reactivity to T22 was found to be driven by the CDR loops of the TCR as exemplified by a Vγ4-Vα11.3/Vδ10 clone, termed G8 TCR (Figure 2a) [48]. The resultant structure was unlike any other, here the G8 TCR bound the collapsed MHC-like antigen presenting groove of T22 with an interface dominated by the δ-chain which corresponded to ∼90% of the buried surface area (BSA) and included key germline encoded contacts (Figure 2f,k) [49]. This structure revealed that γδ T cells could adopt highly unconventional docking strategies in mice although the translation to humans and other antigens was unknown.

The clearest insight we have to date on human γδ T cell reactivities centres on adaptive Vδ1 T cells responding to MHC-like molecules. A population of peripheral CD1d restricted Vδ1^+^ population of T cells found to exhibit differing antigens specificities and representing 0.05–3.5% of CD1d–tetramer^+^ cells were isolated. Here an element of CD1d autoreactivity was identified with ligand independent tetramer reactivities although some clones showed differing lipid specificities α -galactosylceramide (α-GalCer), β-galactosylceramide (β-GalCer) and β-glucosylceramide (β-GlcCer). Notably the phenotype and functional potential of the CD1d restricted γδ T cells differed from a type I NKT (CD8^−^/CD4^−^, levels of IFN-γ production). One specific clone, termed 9C2 (Vγ5–Vδ1^+^) was shown to be strongly responsive to CD1d presenting α-GalCer but some autoreactivity to CD1d [37]. Indeed, a resultant structure provided molecular insight into this autoreactivity, with the 9C2 TCR binding the polar extreme of the CD1d A’ pocket, distinct from type I and type II NKT TCR–CD1d recognition (Figure 2b) [50,51]. The interface comprised of a majority (75%) of δ-chain contacts which included a mix of germline and non-germline contacts to CD1d whereas the ligand mediating contacts involved the CDR3γ (25% BSA γ-chain) (Figure 2g,l).

Further insight into this T cell niche was afforded upon the isolation of a γδ T cell population responsive to CD1d presenting sulfatide, a sulfated β-galactosylceramide that is abundant in myelin and associated in demyelination diseases [52]. Here a rarefied population, 1% of peripheral Vδ1^+^ T cells, of CD1d-sulfatide restricted γδ T-cells was insufficient to enable phenotyping although one clone termed DP10.7 (Vγ4Vδ1), was shown to confer sulfatide specificity [38]. Here the DP10.7 TCR docked similarly to the 9C2 clone, but closer to the type II NKT αβ counterpart (Figure 2c) [51]. As with the similar docking manner, the interface was biased towards δ-chain contacts here accounting for the entire interface (Figure 2h,m) [38]. As observed with 9C2, germline encoded regions mediated contact to the CD1d surface however for DP10.7 the CDR3δ provided the ligand specific contacts to the presented lipid antigen. Thus, the Vγ played no role in TCR recognition of CD1d-sulfatide and only ligand deciphering contacts to CD1d presenting α-GalCer.

Following the isolation of a Vδ1^+^ CD1d γδ subset, an MR1 reactive peripheral blood and tissue γδ T cell population was identified. These cells ranged from <0.1–5% of γδ T cells and were mostly CD8^−^/CD4^−^ with variable CD161 expression, in contrast with typical MR1-restricted αβ T cells. Many of the responding γδ T cell clones displayed varied activation thresholds and antigen specificity with suggested auto reactivity. A clone termed G7 (Vγ9Vδ1) bound MR1 with moderate affinity whether presenting 5-(2-oxopropylideneamino)-6-D-ribitylaminouracil (5-OP-RU), 6-formylpterin (6-FP) or acetyl-6-formylpterin (Ac-6-FP). Structural analysis of this clone revealed a completely novel docking topology binding the underneath of the MR1 antigen presenting groove, unlike any γδ TCR (Figure 2e). Indeed, the interaction was beyond anything observed for MR1 reactive αβ T cells which converge on the antigen presenting groove following conventional paradigms.

Despite such a novel interaction with MR1, the interface was directed by the δ-chain which comprised 84% of the BSA (16% γ-chain) (Figure 2i,n). The δ-chain contacts to the α3 domain of MR1 was principally germline encoded, including the CDR1δ, δ-chain framework residues alongside some non-germline CDR3δ contacts. Thus, the building picture of γδ TCR mediated reactivity is one where the δ-chain plays a principle role, indeed mutagenesis studies of MR1 to disrupt the paratope of the δ-chain showed both its central role in the docking mechanism employed by the G7 TCR, and showed other clones converged on neighbouring regions of MR1.

The exception to this evolving picture stemmed from *ex vivo* culturing of haematopoietic stem progenitor cells to yield MART-1-HLA*A2 reactive CD8^+^ αβ and γδ T cells, with analogous populations derived from CD4^−^ T cells from umbilical cord blood. Functional testing was limited due to the scarcity of such cells however MART-1–specific γδ TCR transduced cell lines produced interferon-γ and displayed effector function after stimulation, expressing granzyme B) and CD107a. Structural insight into one clone, termed 5F3 was structurally resolved to reveal an unorthodox docking mode again. Here using a δ-α comparisons the 5F3 TCR docked over the antigen binding groove although in reverse orientation, when compared with an αβ restricted MART-1 HLA*A2, [53] akin to a few αβ exceptions (Figure 2d) [54,55]. In separation from other γδ structures the 5F3 had a relatively balanced interface, whereby the γ- and δ-chains contributed equally (59 & 41% BSA, respectively) to the interaction (Figure 2j,o). The interface included contacts from each CDR loop, except for the CDR3γ, although a key reactivity conferring interaction stemmed from W98 of the CDR3δ. Here W98 made prominent contacts to the MART-1 peptide replicating analogous peptide contacts from a key hydrophobic observed in αβ restriction. Whilst questions remain around the generality of such melanoma specific T cells and their existence in the periphery, this work illustrates that γδ T cells can expand and fill immunological voids as seen in diseases that deplete MAIT cells [56].

Thus, in conclusion, the paradigms that have held for αβ restriction to MHC over decades are yet to hold for any determined γδ TCR mediated antigen recognition pairing. Instead the emergent theme is one whereby each TCR adopts a recognition mechanism unique to the clone, or a small sub-population of the responding cells. The antigen recognition interfaces include a dominant role of the δ-chain, sometimes exclusively, leaving the γ-chain either to play an ancillary role in ligand deciphering or completely free from the interface. Early studies have noted that the CDRδ chain is the most polymorphic and diverse, correlating with its ability to endow such varied docking modes, however the role of the γ-chain remains ill-defined, at least in an adaptive capacity.

## The functional role of Vγ gene segments in BTN/L reactivity

Intriguingly, the γ-chain is of central importance in some innate-like γδ T cell subsets, whereby strong restrictions of the Vγ gene usage correlate with TCR mediated reactivities to infectious disease or tumour associated metabolites. Namely, Vγ9Vδ2 cells were shown to respond to non-peptide pAg (IPP & HMPP) [57–61]. Since these seminal discoveries, it was found that such γδ T cells are activated in a Vγ9Vδ2 TCR dependant manner that is dependent on members of the butyrophilin 3A family (BTN3A1, BTN3A2, BTN3A3) [62,6344]. Structural insight into the BTN3A molecules revealed the architecture of the extracellular IgV and IgC domains which provided insight into homodimerization [64].

**Figure 3.**
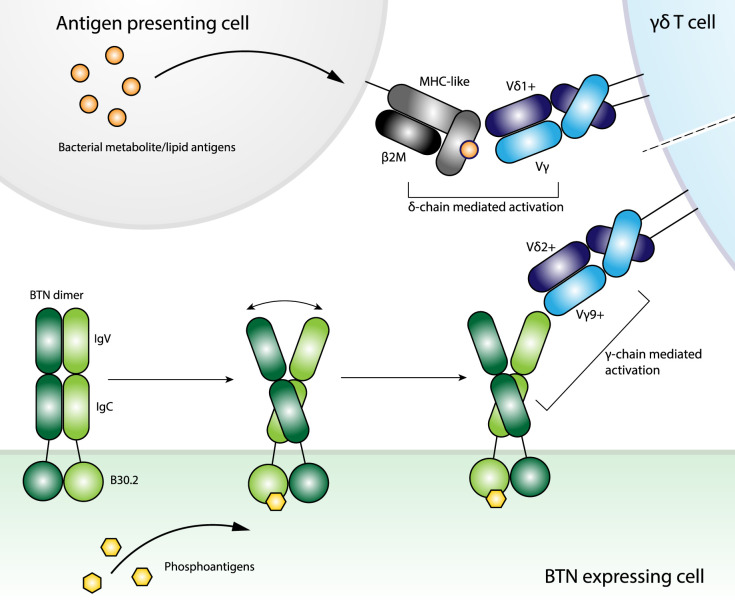
The divergent activation pathways of adaptive-like and innate-like γδ T cells. The activation mechanisms of adaptive-like γδ T cells, so far, centre of Vδ1 mediated recognition of MHC-like molecules employing a heavy Vδ-dominance for cognate antigen ligation. Innate-like γδ T cells recognise bacterial lipid antigens (HMBPP & IPP) in a butyrophilin dependent manner and display a restricted Vγ gene pairing. Although the molecular basis remains unknown Vγ9^+^ and Vγ4^+^ TCR confer reactivity to BTN3A1 and BTNL3, respectively, thus implicating the γ-chain at the interface.

Contention arose over how the pAg induced BTN3A mediated Vγ9Vδ2 activation was achieved. Initial suggestion of a possible antigen presentation role for BTN3A molecules is now viewed as unlikely due to mounting evidence of the central role of the B30.2 domain in pAg binding [65]. Structures of the B30.2 domain of BTN3A1 in complex with pAgs showed the ligand binds within a positively charged pocket, [46,66] in close agreement with mutational studies of the binding site, [67] with binding shown to induce conformational changes within the B30.2 domain [68]. Furthermore, this study suggested that BTN3A1 formed a V-shaped homodimer on a resting cell-surface, with di-sulphide locking of the homodimer shown to impede pAg detection [68]. Recently, BTN2A1 has been implicated in pAg reactivity by Vγ9Vδ2T cells. Here, BTN2A1 expression was closely associated with BTN3A1 at the cell-surface with co-expression required for pAg reactivity, suggesting a potential role for heterodimerisation although direct evidence of this is outstanding despite the observation of BTN3A1 heterodimers [47,69]. Furthermore, BTN2A1 was shown to directly bind a charged patch of the TCR in a germline encoded region of the Vγ9, distal to the CDR loops that was found to be central to the reactivity [47]. Thus the evolving picture of Vγ9Vδ2 reactivity to pAgs involves BTN3A1 & BTN2A1 which may heterodimerise and then transmits an extracellular signal or conformational change upon intracellular binding of pAg binding via the B30.2 domain (Figure 3). However, recent identification of BTN3A1 interacting with CD45-phosphatases, provides an alternative hypothesis, that pAg binding BTN3A1 then sequesters CD45 from the immune synapse allowing an as yet unidentified CDR3-dependent antigen to be recognised by the Vγ9Vδ2 TCR [70].

A tissue restricted population of Vγ4^+^ γδ T cells have been found to display reactivity to another butyrophilin family member, the butyrophilin-like (BTNL) molecule BTNL3, which heterodimerises with BTNL8 and resulted in a TCR-dependent response [69,71,72]. Indeed BTNL3 recognition by Vγ4^+^ γδ T cells led to CD69 up-regulation and TCR internalisation [71]. Sequence comparisons of BTNL3 reactive and non-reactive clones in conjunction with mutational studies of the TCR revealed key roles for germline encoded CDR2γ and hypervariable region 4, termed HV4. Furthermore, characterisation of a soluble Vγ4^+^ TCR termed LES, was shown to directly bind the IgV domain of BTNL3 with moderate affinity (∼15–25 µM) with mutations of the CDR2γ and HV4 region mitigating BTNL3 reactivity or conferring responsiveness upon inclusion of such motifs within typically non-responsive γδ TCRs [71]. Mutational analysis of the BTNL3 molecule suggested that the CFG face of the IgV severely impacted binding by the LES TCR [71]. The mutational work led to a proposed model for the potential interaction although atomic structural data on this front is currently lacking.

Further work on IEL gut resident Vγ4^+^ TCRs suggested a contesting hypothesis of BTNL3 restriction in celiac patients and are implicated in the inflammatory response therein [73]. Here single cell sequence analysis of the Vδ1^+^IEL TCR repertoires identified a CDR3γ histidine motif neighbouring the TRGJ1-encoded Jγ segment (termed H-J1) that was overrepresented in patients with active celiac relative to healthy controls and celiac patients with gluten-free diets [73]. Further investigation showed remodelling of the IEL γδ T cell compartment within celiac patients with altered ability to respond to BTNL3/8 correlated with disease progression. The underlying mechanism and the importance of the H-J1 motif are still to be determined with a large scale single-cell study of γδ TCR sequences from celiac patients unable to identify the H-J1 motif [74].

Thus, Vγ4^+^ γδ T cells directly respond to BTNL3 in isolation from BTNL8, although they are thought to heterodimerise prior to cell surface expression [69]. Contesting hypotheses currently exist over the molecular basis for such restriction with elegant cellular work implicating either the germline HV4 regions or the non-germline H-J1 TCR regions of the γ-chain.

## Discussion

The relative abundance of bona fide γδ T cell ligands although threadbare has yielded pivotal insight into the molecular determinants of these enigmatic cells. What has become clear is that the paradigms of conventional T cell restrictions do not translate. Instead each subset must be treated individually despite the desire for universal truths. One general trend is the Vδ1-chain to dictate much of the interface in adaptive γδ T cell responses, at least thus far. Such δ-chain dominance, in combination with the unique and extreme polymorphic capacity of this chain, [75] has enabled extremely unorthodox T cell recognition mechanisms that resemble those observed for antibody mediated B cell immunity (Figure 3).

In contrast with the δ-chain dominance of the adaptive antigen driven responses, a subset of innate-like γδ T cells show a strong Vγ restriction. Here peripheral Vγ9^+^ γδ T cells confer reactivity to foreign lipid antigens in a BTN3A1 and BTN2A1 dependent manner. Whereas, Vγ4^+^ IEL γδ T cells respond to the BTNL3 and BTNL8 heterodimer although the biological context for this interaction and any disease associated triggers remains unclear. Comprehensive mutational work has identified regions within the Vγ domain that confers BTN/L reactivity and map to a site distinct from the antigen binding paratope of their adaptive counterparts (Figure 3). Notably due to the δ-chain dominance described above, the BTN/L binding regions of the Vγ domain are typically accessible in adaptive antigen-driven complexes. Thus, one of the central unanswered questions of such innate-like γδ T cell biology is the basis for the Vγ restrictions in both BTN and BTNL mediated axes of γδ T cell activation. Indeed, structural clarity on this front will be particularly revealing.

The importance and function of defined γδ T cell interactions with cognate ligands within disease settings is not well understood. An expanded understanding of the potential ligand repertoire of γδ T cells will likely serve as useful tools in deciphering such immune roles within infections and cancer. A tantalising premise is the potential convergence of the adaptive and innate capacities of such γδ T cells, namely are antigen recognition and BTN and BTNL ligation events mutual exclusive or compounding. Previous investigations with MHC or B2M deficient cell-lines suggested that MHC and MHC-like molecules are not required although another protein is likely involved in γδ T cell activation [76,77]. Thus, antigen identification experiments and structural analyses of γδ T cells have provided profound insight into their biological functional and unique ligand bindings properties. However, central questions remain about their activation mechanisms, the true diversity of potential antigens and the adaptive or innate differences in γδ TCR ligation. Answering of these questions will aid in our understanding of γδ T cells and unlock their therapeutic potential moving forward.

## Perspectives

- γδ T cells comprise a central yet often overlooked component of the tripartite adaptive immune system with particularly key roles in epithelial immunity and protection.
- Current knowledge on the activating ligands and recognition principles of γδ T cells are incomplete. Despite this γδ T cells are already being developed within the context of cellular immunotherapies.
- To fully realise the importance of γδ T cells in protective immunity, and unleash their true immunotherapeutic potential, we must decipher the breadth of antigens that γδ T cells can recognise and elucidate the activation paradigms across γδ T cell subtypes.

## Open Access Statement

Open access for this article was enabled by the participation of Monash University in an all-inclusive Read & Publish pilot with Portland Press and the Biochemical Society under a transformative agreement with CAUL.

## Abbreviations

5-OP-RU: 5-(2-oxopropylideneamino)-6-D-ribitylaminouracil
6-FP: 6-formylpterin
Ac-6-FP: acetyl-6-formylpterin
BTN: Butyrophilin
BTNL: Butyrophilin-like
HMBPP: (E)-4-Hydroxy-3-methyl-but-2-enyl pyrophosphate
IPP: isopentenyl pyrophosphate
TCR: T cell receptor
termed H-J1: histidine motif neighbouring the TRGJ1-encoded Jγ segment
α-GalCer: α-galactosylceramide
β-GalCer: β-galactosylceramide
β-GlcCer: β-glucosylceramide
